# Adapting the supervisory relationship measure for general medical practice

**DOI:** 10.1186/s12909-018-1369-x

**Published:** 2018-11-27

**Authors:** Shane Costello, Rebecca Kippen, Joan Burns

**Affiliations:** 1 Faculty of Education, Monash University, 19 Ancora Imparo Way, Clayton, 3800 Australia; 2 School of Rural Health, Monash University, Sydney, Australia; 3 GP Supervisors Australia, University of New South Wales, Sydney, Australia; 4 School of Public Health and Community Medicine, University of New South Wales, Sydney, Australia

**Keywords:** Supervisory relationship, Educational alliance, General practice, Clinical education

## Abstract

**Background:**

The relationship between general practice (GP) supervisors and registrars is a critical component in effective training for the next generation of medical practitioners. Despite the importance of the relational aspect of clinical education, most evaluation has traditionally occurred from the perspective of the registrar only. As such, no validated tools exist to measure the quality of the supervisory relationship from the perspective of the supervisor. This paper presents an adaptation and validation of the clinical psychology supervisory relationship measure (Pearce et al, Br J Clin Psychol 52:249–68, 2013) for GP supervisors in an Australian context.

**Method:**

Following an Expert Group review and adaptation of the items, 338 GP supervisors completed the adapted tool.

**Results:**

Using principal components analysis and Procrustes confirmatory rotation, an optimal three-component model of supervisory relationship was identified, reflecting measures of Safe base (*α* = .96), Supervisor investment (*α* = .85), and Registrar professionalism (*α* = .94).

**Conclusions:**

The general practice supervisory relationship measure (GP-SRM) demonstrated excellent model fit, high internal consistency, and was theoretically consistent with the original tool. Implications for clinical education and future research are presented.

## Background

General practice (GP) supervisors are critical to the training of the next generation of general practitioners. GP supervisors provide “guidance and feedback on matters of personal, professional and educational development in the context of a trainee’s experience of providing safe and appropriate patient care” [1]. The feedback that GP supervisors provide to registrars is recognised as a critical component in Australian specialist medical education [2] and is explicitly part of the Australian College of Rural and Remote Medicine [3] and the Australian Medical Council Specialist Education Accreditation Committee [4] supervision standards for general practice training. Yet, despite this importance, little attention is paid to the experiences of GP supervisors, and in particular to the experiences of the educational relationship between supervisors and registrars.

Supervisor satisfaction is a critical motivator to engage and retain supervisors in clinical and educational supervision [5, 6]. The quality of the relationship between supervisors and registrars underpins supervisor satisfaction. Despite this, most studies of the educational environment, clinical learning environment or educational alliance have tended to focus on the relationship from the perspective of the registrar only [7–10].

Registrar perceptions of their training experience are used as part of the quality assurance process for accrediting anaesthetic training programs in the UK [11]. In the USA, the Clinical Learning Environment Review was introduced by the Accreditation Council for Graduate Medical Education to provide feedback to hospitals and medical centres on how successful they are at engaging residents to improve quality and safety systems in the clinical learning environment [12].

The relationship between a registrar and their supervisor has been suggested as the platform for all other aspects of learning. The concept of educational alliance is emergent in the literature as a central component of supervision and commonly included in definitions used for GP supervisors. Wearne et al. [13] described an effective supervisor as “a general practitioner who establishes and maintains an educational alliance that supports the clinical, educational and personal development of a resident” (p. 1169). The Australian General Practice Training (AGPT) program describes GP supervisors as “experienced general practitioners [who] establish and maintain educational alliances that support the clinical, educational and personal development of registrars who come to work in their practices”. (AGPT [14];).

Yet evaluation of this relationship, or alliance, is entirely focused on registrar satisfaction with the supervision and training they received. There are no measurement tools available that capture the perceptions and experiences of the GP supervisor in their relationship with supervised registrars. Evaluating the educational alliance from the point of view of the GP supervisor holds great potential as a feedback framework [15] and thereafter a measure and predictor of supervisor and registrar satisfaction, and supervisor retention, providing important insights into the type of supervisor educational supports required for junior supervisors joining GP training.

In other disciplines, tools have been developed to measure supervisory relationships from the point of view of supervisors. For example, a study of the perception of medical faculty members of their educational environment in teaching undergraduate medical students led to the development a 50-item inventory, the Assessment of Medical Education Environment by Teachers [16]. Clinical psychology is a field where the relationship between supervisor and registrar is considered so important that its formation and management is included as a core competency internationally [17].

Reconsideration of the role of the supervisor in providing feedback within the context of the educational alliance is also suggested by others [18]. It has been proposed that the field of medical education could benefit by examining the therapeutic alliance (the relationship between supervising psychologists and trainee psychologists, which is a significant mechanism by which positive outcomes are achieved) in psychotherapy training as analogous to the educational alliance in medical training, in particular the use of feedback [15]. This perspective is one which offers an innovative framework from which to reconceptualise feedback and suggests that new research questions should explore the educational alliance between registrars and supervisors, particularly in other disciplines.

Despite the lack of tools to measure the supervisory relationship in medical education, there is one validated tool designed for use in a parallel context. The Supervisory Relationship Measure (SRM) is a 51-item questionnaire which was developed as a measure of the supervisory relationship between clinical psychology supervisors and their registrars, from the perspective of the supervisor [19]. In the original SRM development study, principal components analysis identified five aspects of supervisor experience and perceptions: “Safe base”, “Supervisor commitment”, “Trainee contribution”, “External influences”, and “Supervisor investment” [19]. The SRM was demonstrated to be a valid and reliable measure of the supervisory relationship from the supervisor’s perspective. A recent independent review of supervisory relationship measures found that the SRM is “a sound measure of the supervisory relationship”, with a large sample used for initial verification, and application of several tests for validity and reliability [20].

While a number of studies have examined registrar satisfaction with supervision, none have attempted to measure the GP-supervisor perspective of the relationship. The current study addresses this dearth of research by adapting and implementing an existing reliable and validated tool to identify key relationship deficits and professional development opportunities for GP supervisors within the Australian context.

## Method

In this study, we adapted and validated the Supervisory Relationship Measure (SRM) for use with GP supervisors. The original SRM, developed for use with clinical psychology supervisors [19], consists of seven-point Likert-scale items which measure the level of agreement (strongly disagree to strongly agree) with 51 statements regarding the supervisory relationship with a particular clinical trainee, such as “*My trainee and I have a good professional relationship”* and “*My trainee is open about any difficulties they are experiencing*”. Reversed responses for statements asked in the negative, such as “*My registrar has a poor professional approach*” were recoded for consistency. Where used, the phrase “trainee” was replaced with “registrar”, which is a more appropriate term in the GP training context. An Expert Group reviewed and adapted items, which was followed by pilot testing. Ethical approval for the study was provided by the Monash University Human Research Ethics Committee (project number 10977).

### Expert group review and pilot testing

The 51 original SRM items [18] were assessed by an Expert Group of four experienced GP Supervisors. These supervisors were recruited from General Practice Training Tasmania. The Expert Group were current supervisors who had between three and 20 years’ experience as a supervisor, and had supervised six or more registrars. Through iterative expert consensus [21] the Expert Group determined the appropriateness of each item, adapted items when appropriate, deleted inappropriate or irrelevant items, and generated new items for use with GP supervisors, creating the GP-SRMS.

Most items were deemed appropriate for retention. The Expert Group made only minor changes to these items; for example, replacing “trainee” with “registrar” and “placement” with “practice term”.

Six items were removed:

1. *My trainee is open to new experiences on placement*
2. *I set up regular supervision for my trainee*
3. *My trainee produces good quality work*
4. *My trainee’s past experiences of supervision interfere with our relationship*
5. *I sense that my trainee worries because I am evaluating them*
6. *My trainee is too anxious to engage in supervision*

When electing to remove items, the Expert Group considered a number of criteria. Items which were not considered accurate in the GP training context included reference to regular supervision (being generally provided less regularly), or concerns around evaluation (because evaluation was unavoidable). The Expert Group considered items relating to a registrar being open to new experiences, past experiences, or mood to be unrelated to the educational alliance. Five new items were added by the Expert Group:

1. *I’m enthusiastic about my registrar’s practice term with me*
2. *I provide the environment and opportunities for my registrar to give me open and honest feedback*
3. *My registrar is not clinically competent*
4. *My registrar’s practice is safe*
5. *My registrar’s self-directed learning interferes with their clinical practice.*

These adapted items, along with questions on demographic characteristics of the supervisor and registrar, were piloted in an online survey using SurveyMonkey. The pilot testers were six GP supervisors with a range of supervisory experience, all recruited from General Practice Training Tasmania. Those participating in the pilot gave written feedback on item clarity and appropriateness, and time taken to complete the survey. One of the aims of the pilot testing was to estimate typical completion time, with the aim to keep completion time less than 15 min, for higher response rates [22]. Five of the six pilot testers stated that they completed the survey in 10–15 min, with only one taking longer - a reported 25 min. All stated that they found all the items clear and appropriate. Thus, no further changes were made to the survey.

### Data collection

Once the survey items were finalised, GP Supervisors Australia sent an email to all 3200 members inviting them to participate in a short anonymous online survey on the supervisory experience, provided they had supervised at least one GP Registrar in the previous 3 months. The email included the aim of the survey, estimated completion time, and a link to the survey conducted through SurveyMonkey. There were 365 GPSA members who answered the GP-SRMS survey demographic questions and a slightly smaller subset of 338 who answered the demographic questions and all 50 GP-SRMS items (11% response rate). Overall, the mean missing data rate across the GP-SRMS items was 5.50%, ranging from 0.27 to 8.28%. The validation was conducted on the 338 participants who provided complete answers.

### Demographic characteristics of survey participants

Demographic characteristics of survey supervisor participants and their registrars are shown in Table 1. The median and modal age group of supervisors responding to the survey was 50–54 years, with ages ranging from 30 to 34 years to 65+ years. Just over half of survey participants were male. Around half had been supervising registrars for more than 10 years, and just over 40% had supervised ten or more registrars. One-quarter were the sole supervisor of the registrar that was the subject of the SRM items, 55% were primary supervisor, and 20% were the secondary supervisor in a group of supervisors. Of registrars who were the subject of the SRM items, half were aged 25–29 years, with another 29% aged 30–34 years. Sixty per cent were female and 42% were at the first level of training.

**Table 1:** Demographic characteristics of GP-SRMS supervisor-participants and their registrars

Supervisor characteristics	% All participants	% Answered all 50 items	Registrar characteristics	% All participants	% Answered all 50 items
Supervisor age			Registrar age		
30–34 years	4.1	3.8	Under 25 years	2.5	2.7
35–39 years	6.8	6.5	25–29 years	50.4	50.9
40–44 years	10.7	10.4	30–34 years	29.0	29.0
45–49 years	14.5	14.8	35–39 years	7.9	7.7
50–54 years	20.8	21.0	40–44 years	7.1	6.8
55–59 years	19.7	20.4	45+ years	3.0	3.0
60–64 years	15.6	15.4	Total	100.0	100.0
65+ years	7.7	7.7			
Total	100.0	100.0			
Supervisor gender			Registrar gender		
Male	51.8	52.7	Male	39.7	39.1
Female	47.7	46.7	Female	59.7	60.4
Other/Prefer not to say	0.5	0.6	Other/Prefer not to say	0.5	0.6
Total	100.0	100.0	Total	100.0	100.0
Time as supervisor			Registrar level of training		
0–1 years	8.2	8	GPT1/PRRT1	41.9	43.2
2–4 years	21.9	21.3	GPT2/PRRT2	15.1	14.8
5–9 years	20.5	20.7	GPT3/PRRT3	28.5	27.8
10–19 years	28.2	28.4	GPT4/PRRT4	12.9	12.7
20+ years	21.1	21.6	Other	1.6	1.5
Total	100.0	100.0	Total	100.0	100.0
No. of registrars supervised as primary/lead			Supervisor relationship to registrar		
0–1	14.2	13.9	Sole supervisor	24.7	24.9
2–4	20.3	20.1	Primary supervisor in	55.1	55.3
5–9	17.8	17.5	a group of supervisors		
10–19	15.3	16.0	Secondary supervisor in	20.3	19.8
20+	27.1	27.5	a group of supervisors		
Not given	5.2	5.0	Total	100.0	100.0
Total	100.0	100.0	**N**	**365**	**338**

Bold font indicates primary component loading

### Statistical analysis

Building on the previous statistical validation of the SRM, principal components analysis (PCA) with Procrustes transformation [23, 24] was used. This method has been described as a confirmatory PCA and provides a measure of model fit. The Procrustes transformation compares the rotated solution to an ideal matrix where items either load completely or not at all; providing an estimate of how well items fit. Using SPSS version 24 [25] PCA with direct oblimin rotation was conducted with all GP-SRMS items, followed by Procrustes transformation using Orthosim version 2.01 [26]. Items with low communality, low primary loading or significant cross-loading, or poor fit were systematically removed until a stable component structure and robust model fit was achieved.

## Results

A Kaiser-Meyer-Olkin value of .95 [27, 28] and a significant Bartlett’s Test of Sphericity [29] were found, supporting the factorability of the correlation matrix. While eight components had eigenvalues exceeding 1, initially five components were extracted consistent with the factor structure of the SRM. A review of the model using Cattell’s scree test [30] and Parallel Analysis [31] suggested that the five component model was an overextraction, with three or four components being more appropriate.

Three, four, and five component models of the GP-SRMS items were examined, and poor items were systematically removed. An optimal model fit was achieved with a three component model which retained 45 items. The overall solution congruence with an ideal target matrix was .97, with values of .85 and above indicating similarity [32, 33]. Two additional measures of congruence were also calculated, with the Double-Scaled Euclidean Distance (.90) and the Kernel Smoothed Distance (.84) both indicating similarity [34, 35]. Component fit was also very high. The final principal components loading matrix, model fit statistics, and reliability coefficients can be found in Table 2.

**Table 2:** Principal Components Analysis (PCA) of GP-SRMS items

	Communalities	1	2	3	Item fit
6. My registrar is enthusiastic about being in the practice with me.	.77	**.90**	−.05	.02	1.00
3. There is a good emotional atmosphere in supervision with my registrar.	.72	**.88**	−.11	.01	0.99
4. My registrar is open and honest in supervision.	.80	**.88**	−.10	−.07	0.99
1. My registrar is open about any difficulties they are experiencing.	.65	**.84**	−.02	.06	1.00
10. My registrar seems to like me.	.67	**.81**	.08	.03	1.00
5. My registrar is willing to learn and experience new things.	.70	**.77**	−.11	−.14	0.98
8. My registrar appears able to give me honest and open feedback.	.65	**.76**	.11	−.01	0.99
12. My registrar and I have a good professional relationship.	.74	**.74**	−.01	−.19	0.98
2. My registrar is reflective in supervision.	.62	**.73**	−.05	−.12	0.99
7. I am enthusiastic about my registrar’s practice term with me.	.66	**.65**	−.01	−.23	0.97
18. My registrar values my experience and skills.	.64	**.64**	.11	−.17	0.97
11. I like my registrar.	.68	**.61**	.07	−.27	0.96
16. My registrar is open minded and curious.	.65	**.60**	.06	−.26	0.96
17. My registrar’s style and my own style interact well.	.64	**.49**	.10	−.36	0.92
13. Supervision provides a safe space for my registrar to learn.	.48	**.44**	.36	−.07	0.88
49. I feel safe giving my registrar negative feedback.	.31	**.42**	.26	.00	0.91
50. I have a good idea about what my registrar wants to gain from this practice term.	.38	**.36**	.33	−.10	0.86
25. I attempt to facilitate reflection in my supervision with my registrar.	.51	−.10	**.74**	.02	0.99
20. I keep my registrar’s needs in mind.	.54	.00	**.70**	−.10	0.99
26. I give clear and honest feedback to my registrar.	.51	.07	**.69**	.02	0.99
21. I try to ensure my registrar has adequate space and resources.	.43	−.07	**.67**	−.03	1.00
22. I am prepared for my registrar prior to their practice term.	.41	−.01	**.65**	.07	1.00
24. I look out for clinical work and other opportunities for my registrar.	.41	.08	**.63**	.08	0.99
23. I am available and accessible to my registrar.	.34	.00	**.60**	.06	1.00
46. I am open in supervision with my registrar.	.39	.09	**.56**	−.09	0.96
19. I try to pitch things at the right level for my registrar.	.33	−.05	**.55**	−.14	0.98
9. I provide the environment and opportunities for my registrar to give me open & honest feedback.	.47	.43	**.54**	.30	0.86
47. I try to get to know my registrar.	.28	.00	**.52**	.00	1.00
48. I am able to share my strengths and weaknesses with my registrar.	.26	−.15	**.51**	−.12	0.97
45. I am aware of what interests my registrar.	.26	.06	**.44**	−.11	0.95
37. I am disappointed by my registrar’s level of skill.	.68	−.03	−.07	**.79**	1.00
30. My registrar copes with multiple demands.	.72	.06	.08	**−.78**	1.00
32. My registrar shows good organisational skills.	.68	.07	.03	**−.78**	1.00
27. My registrar is able to hold an appropriate case load.	.62	−.05	.16	**−.77**	0.99
29. My registrar works hard in the practice.	.60	−.04	.13	**−.76**	0.99
28. My registrar appears to be doing only the minimum expected.	.48	.02	.09	**.72**	0.99
14. My registrar is not clinically competent.	.37	−.01	.03	**.61**	1.00
33. My registrar has a poor professional approach.	.49	−.16	.05	**.60**	0.99
34. My registrar takes appropriate responsibility for their work.	.58	.27	−.06	**−.58**	0.97
36. My registrar is appropriate in their interprofessional communication.	.72	.40	.03	**−.54**	0.93
35. My registrar integrates well with others in the team.	.75	.42	.03	**−.53**	0.93
15. My registrar’s practise is safe.	.53	.25	.09	**−.52**	0.96
38. I value having my registrar in the practice.	.51	.23	.19	**−.47**	0.93
31. My registrar is considerate towards others in the practice (e.g. all practice staff).	.64	.46	−.08	**−.46**	0.90
44. Evaluation of my registrar’s performance has a negative impact on our relationship.	.38	−.31	−.03	**.37**	0.92
	Component fit	0.99	0.94	0.86	
	Cronbach’s α	.96	.85	.94	

Items reproduced with permission. The GP-SRMS can be freely accessed online [37].

Bold font indicates primary component loading

## Discussion

The aim of the current study was to adapt and validate the SRM for use with GP supervisors within the general medical practice training context. The relationship between clinical supervisors and registrars has been demonstrated to be vital in fields such as clinical psychology [19], and it was our contention that the therapeutic alliance between supervisors and trainees in psychology was analogous to the educational alliance in medical training [17–19], highlighting the need to consider supervisory relationships in medical training.

In the original SRM development study, principal components analysis identified five aspects of supervisor experience and perceptions. The results of the current study suggested that a three component model comprising “Safe base”, “Supervisor investment”, and “Registrar professionalism” was more appropriate in the general medical practice training context, following the removal of several poorly performing items. Building on the statistical methodology which was used to develop the SRM, the three component GP-SRMS demonstrated excellent model fit overall, as well as within components. The original SRM demonstrated subscale reliabilities ranging from .71 to .96. The GP-SRMS subscales matched or exceeded the reliabilities achieved by the SRM, ranging from .85 to .96. The fourth SRM subscale “External influences” and fifth SRM subscale “Supervisor investment” likely represent an overextraction in the original study, which also found some evidence for a two or three component model.

In the original SRM study, the “Safe base” subscale contained the highest number of items and the highest reliability. The final GP-SRMS “Safe base” subscale retained the equivalent items, and also gained two items, one of which had been previously been included with “Supervisor investment” in the SRM. The item “*I feel safe giving my registrar negative feedback”* had no direct equivalent in the SRM, however it was semantically and theoretically consistent with “Safe base”. The item “*I have a good idea about what my registrar wants to gain from this practice term”* could contribute to a registrar feeling valued and understood, which is consistent with other “Safe base” items. Scores on the “Safe base” subscale reflect an enthusiastic, open, collaborative GP supervisory relationship.

The “Supervisor investment” subscale in the GP-SRMS reflects a combination of the items which originally comprised “Supervisor investment” and “Supervisor commitment” in the SRM. The original subscales separated more clearly into professional commitment and emotional investment, and it is possible that the Expert Group item changes reflect a subtle shift away from emotional investment, or that there is less differentiation between professional and emotional aspects of supervision in the medical context. Scores on the “Supervisor investment” subscale reflect a GP supervisor’s effort to support the registrar through resources, preparation, and being interested in the registrar.

“Trainee contribution” in the SRM was largely subsumed into “Registrar professionalism” in the GP-SRMS. While nearly all of the equivalent items were retained, several items such as “*My registrar is not clinically competent”* and “*My registrar’s practice is safe*” and the Expert Group item changes reflect a more explicit focus on professionalism rather than contribution in the broader sense. Scores on the “Registrar professionalism” subscale reflect a GP supervisor’s perceptions of how competent, responsible, organised, and committed a registrar is.

The “External influences” subscale of the original SRM did not emerge clearly in any of the component models of the GP-SRMS. The poorly fitting item “*My registrar wants me to be their GP as well as their supervisor*” perhaps reflects a difference in the supervisory relationship between general practice medicine and clinical psychology. Alternatively, it is possible that a GP supervisor would not consider a registrar who discloses or seeks out medical consultation to be acting inappropriately. Two items relating to life stressors (“*My registrar has other life stressors which distract them from their work*” and “*I have stressors in my life which make it difficult for me to focus on supervision*”) did not demonstrate adequate model fit, which suggests that life stressors are typically less of an factor in the GP supervisory relationship, regardless of whether the stressors are experienced by the supervisor or registrar. Finally, two items relating to education and learning (“*My registrar’s educational training requirements interfere with their clinical practice”* and “*My registrar’s self-directed learning interferes with their clinical practice*”) also failed to demonstrate adequate model fit. The latter item was suggested by the Expert Group, however it is possible that this experience in supervision was not as widely encountered, or perceived to be as damaging to the supervisory relationship. It could also be suggested that education and learning requirements will vary between training programs and placements, reducing the consistency in which this aspect is experienced. While we are unable to be certain why “External influences” failed to emerge in the GP-SRMS, there was evidence that a model with more than three components was an overextraction. The results of the current study suggest that the three component model demonstrated better model fit and reliability without this subscale and associated items.

The current study is not without limitations. With a response rate of approximately 10%, there remains a question about the representativeness of the sample used. However, there is evidence to suggest that response-rate bias is not overly problematic for most quantitative analyses, suggesting that the results are robust [36]. GP supervisor selection of registrars was also not randomised or stratified, which may have impacted on the distribution of responses. Test-retest reliability has not yet been established, nor the convergent validity between GP supervisor reports and GP registrar reports, however this research is currently ongoing.

## Conclusions

The aim of the current study was to adapt and validate the Supervisory Relationship Measure for use with general practitioner supervisors. Following an expert review and psychometric evaluation, the revised GP-SRMS demonstrated excellent psychometric properties across three domains of “Safe base”, “Supervisor investment”, and “Registrar professionalism”. Given the lack of research which considers the supervisory relationship from the perspective of the GP supervisor, the GP-SRMS will be of significant interest to clinical educators. Future research will focus on determining test-retest reliability of the GP-SRMS; convergent validity between supervisor and registrar versions of the tool; and identifying the training needs and guidelines for most effective use of the GP-SRMS in clinical education.

## Abbreviations

AGPT: Australian General Practice Training
GP: General practice or general practitioner
GP-SRMS: General Practice Supervisory Relationship Measure for Supervisors
PCA: Principal Components Analysis
SRM: Supervisory Relationship Measure
